# Efficiency analysis of primary healthcare facilities in Afghanistan

**DOI:** 10.1186/s12962-022-00357-0

**Published:** 2022-06-03

**Authors:** Farhad Farewar, Khwaja Mir Ahad Saeed, Abo Ismael Foshanji, Said Mohammad Karim Alawi, Mohammad Yonus Zawoli, Sinai Irit, Wu Zeng

**Affiliations:** 1 Formerly Ministry of Public Health, Kabul, Afghanistan; 2 Freelance Health Financing Consultant, Kabul, Afghanistan; 3 The Palladium, Washington, DC USA; 4 Department of International Health, Georgetown University, Washington, DC USA; 5 Present Address: Ministry of Public Health, Kabul, Afghanistan

**Keywords:** Relative technical efficiency, Primary healthcare, Data envelopment analysis

## Abstract

**Background:**

Afghanistan’s health system is unique in that primary healthcare is delivered by non-governmental organizations funded by multilateral or bilateral donors, not the government. Given the wide range of implementers providing the basic package of health services, there may be performance differences in service delivery. This study assessed the relative technical efficiency of different levels of primary healthcare services and explored its determinants.

**Method:**

Data envelopment analysis was used to assess the relative technical efficiency of three levels of primary healthcare facilities (comprehensive, basic, and sub-health centers). The inputs included personnel and capital expenditure, while the outputs were measured by the number of facility visits. Data on inputs and outputs were obtained from national health information databases for 1263 healthcare facilities in 31 provinces. Bivariate analysis was conducted to assess the correlation of various elements with efficiency scores. Regression models were used to identify potential factors associated with efficiency scores at the health facility level.

**Results:**

The average efficiency score of health facilities was 0.74 when pooling all 1,263 health facilities, with 102 health facilities (8.1%) having efficiency scores of 1 (100% efficient). The lowest quintile of health facilities had an average efficiency score of 0.36, while the highest quintile had a score of 0.96. On average, efficiency scores of comprehensive health centers were higher than basic and sub-health centers by 0.11 and .07, respectively. In addition, the difference between efficiency scores of facilities in the highest and lowest quintiles was highest in facilities that offer fewer services. Thus, they have the largest room for improvement.

**Conclusions:**

Our findings show that public health facilities in Afghanistan that provide more comprehensive primary health services use their resources more efficiently and that smaller facilities have more room for improvement. A more integrated delivery model would help improve the efficiency of providing primary healthcare in Afghanistan.

## Background

Following two decades of war and the resulting collapse of public health institutions, the government of Afghanistan prior to the 2021 Taliban takeover reshaped the healthcare system, including primary healthcare, in 2002. The Basic Package of Health Services (BPHS) was introduced in 2003 to provide an umbrella of priority primary healthcare services. In Afghanistan, BPHS is offered in six standard types of health facilities, ranging from community outreach provided by community health workers in health posts through outpatient care at health sub-centers (SHCs), basic health centers (BHCs), and mobile health teams (MHTs), and comprehensive health centers (CHCs); and for inpatient services at district hospitals (DHs). Different health facilities have varying sets of standard inputs, such as the number and qualification of staffing, that affect both inputs and outputs of service delivery and thus affect the efficiency. However, while primary healthcare in the country is perceived to be donor-dependent, in reality, a large proportion of total health spending is borne directly by households [1].

Since the introduction of BPHS, the coverage of primary care has been improved substantially. With the expansion of primary health services through BPHS, more people can access healthcare. Meanwhile, the funding gaps are widening over time, and improving efficiency in health service delivery has become increasingly critical to address them. To understand where to improve the fiscal space to fund health care systems in Afghanistan, the Health Economics and Financing Directorate (HEFD) of the Afghanistan Ministry of Public Health conducted a fiscal space analysis in 2016 [2] and identified inefficiencies as a key area for increasing fiscal space for health [3]. In an effort to improve accessibility and move towards the sustainable development goal of universal health coverage, the Afghanistan Ministry of Public health is seeking ways to increase the government's share of total health expenditure on one hand, and to spend resources more efficiently on the other hand.

How BPHS has been funded further complicates the efficiency of primary care delivery in Afghanistan. In 31 of the country’s 34 provinces, the services of BPHS have been contracted out to non-governmental organizations (NGOs), funded by a variety of donors (United States Agency for International Development (USAID), the World Bank, and the European Union). In the remaining three provinces, health services are provided directly by the Afghan government under the Strengthening Mechanism Project [4]. Previous research has revealed potential inefficiency in the NGO-run provinces (31) compared to the three provinces in which the Afghan government provides services directly [5]. The struggle to reduce inefficiencies in the health sector is not unique to Afghanistan. International evidence suggests that inefficiency across health facilities in low-income countries is widespread [6–8].

Given the complexity of BPHS delivery and the recent decrease in donor aid, efficiency enhancement has become a priority task for the Ministry of Public Health in Afghanistan. In this study, we investigated the efficiency of three levels of health facilities: CHCs, BHCs, and SHCs, in the 31 Afghanistan provinces operated by NGOs (totaling 1263 facilities) using the Data Envelopment Analysis (DEA) approach to compare the relative technical efficiencies across different facilities.

## Methods

### Setting

Afghanistan is a landlocked country with a population of 38.9 million. The gross domestic product per capita in Afghanistan was $508.9 in 2020, ranking the country among the least developed economies in the World. Afghanistan is also one of the most fragile and conflict-affected countries in the world, experiencing almost uninterrupted conflict for the last thirty years [9].

Providing primary care services has been challenging in the country. Prior to the withdraw of US troops and the Taliban’s taking over the country, the country delivered primary care services through CHCs, BHCs, SHCs, MHTs, and DHs with primarily donors’ financial support. The study focused on three levels of health facilities (i.e., CHCs, BHCs, and SHCs) in the 31 provinces where BPHS was contracted out to NGOs. The selected three types of facilities consume the most resources for BPHS. While DHs also provide BPHS services, the number of DHs is limited compared to CHCs, BHCs, and SHCs. Each level provides services of various complexity and covers different population sizes; hence, requiring different categories and numbers of staffing.

### Data sources

To assess the efficiency of health facilities using DEA, inputs and outputs for providing BHPS need to be specified. In this analysis, we used health expenditure and the number of various personnel as inputs, while using the number of various services provided by health facilities as outputs. Details of inputs and outputs are described in the section of “Variable description”.

We obtained facility health expenditure data from the Expenditure Management Information System (EMIS) which is centrally managed by the Afghanistan Ministry of Public Health. We constructed our input variables using the EMIS indicators on health facility human resource expenditures (e.g., medical, administrative, and supporting staff), capital investments (e.g., equipment, machinery, and tools), and other recurrent costs of operating health facilities and providing health services. On the other hand, Afghanistan Health Management Information System (HMIS) allowed us to construct output variables using data on the number of outpatient visits by type of service received. The HMIS also provided information on the number of different types of personnel employed in each health facility (additional input factors).

Both EMIS and HMIS have been institutionalized in Afghanistan for many years. They are collected quarterly and are considered to be reliable. All data were extracted for the full 2016 calendar year.

Afghanistan has 34 provinces and only 31 of these provinces report to the EMIS. These are the provinces where NGOs operate health facilities. All health facilities in these provinces were considered for analysis. We excluded facilities with ‘outlier’ expenditures—facilities whose expenditure was plus/minus three standard deviations from the mean facility expenditure. This resulted in 1263 facilities in the analysis: 272 CHCs, 571 BHCs, and 420 SHCs. The number of excluded outlier health facilities were: 1 CHC, 11 BHCs, and 10 SHCs.

### Analytic approach

To estimate the efficiency of health facilities, we used DEA, a classic non-parametric approach, to calculate a measure of relative technical efficiency. An advantage of DEA is that it provides considerable flexibility in data selection and can incorporate multiple input and output variables, which can be continuous, ordinal, or categorical. The DEA calculated an efficiency score for each health facility. Intuitively, the efficiency score could be regarded as weighted outputs to the weighted inputs with the following formula.

$$Efficiency\, Score= \frac{\sum\, weighted \,outputs}{\sum \, weighted \, inputs}$$

Mathematically, the efficiency scores were estimated as a ratio of weighted outputs to weighted inputs, where the weights were calculated by the statistical software automatically by maximizing the ratio for each decision-making unit (DMU) under the evaluation while ensuring that the ratio, when applied to other DMUs, would be between 0 and 1. As DEA has been widely described elsewhere [10–13], the derivation of efficiency scores using DEA is not repeated here. There are two approaches to make DMUs to be efficient: one is to improve outputs while the other is to reduce inputs. An efficiency score is called output-oriented efficiency if the calculation is based on improving outputs, and input-oriented efficiency if it is based on reducing inputs. In this study, we calculated input-oriented efficiency with the assumption of variable returns to scale. This approach would help understand potential savings for given outputs.

In this study, we conducted two DEAs. We first calculated separate efficiency scores for the three levels of facilities: SHC, BHC, and CHC (referred to as “separate efficiency scores”). By the scores, we assumed that each type of facility has its own production frontier. With the calculated input-oriented efficiency scores from this analysis, we estimated potential savings, which are the product of health expenditure and the complement of the efficiency scores. DEA estimates an efficiency score for each health facility, ranging from 0 to 1. An efficiency score of 1 means that the health facility has maximum efficiency, while an efficiency score of 0 suggests that the health facility does not produce any outputs. As the efficiency estimation for the three types of facilities used different production frontiers, the efficiency scores could not be compared across different types of facilities. We pooled all three types of facilities together in the second DEA, assuming a single production frontier (referred to as “pooled efficiency scores”). The efficiency scores from the pooled sample allow for comparing efficiency scores across different types of facilities.

## Variable description

For inputs, we included the following indicators: (1) number of clinical personnel reported for the fourth quarter, (2) number of non-clinical personnel, including administrative and supporting staff, reported for the fourth quarter, (3) non-personnel recurrent expenditure (total for the year), and (4) capital expenditure (total for the year). Given that the number of personnel, including clinical and non-clinical personnel had accounted for the staff inputs, their salaries were not included in the recurrent expenditure. The personnel for the fourth quarter were selected because the actual number of existing staff who work for the entire year was well recognized in the fourth quarter. These four indicators capture most of the resources used by health facilities to provide services. While expenditure information in the EMIS is in Afghanistan currency, the Ministry of Public Health makes programmatic decisions in United States Dollars (USD). Therefore, we converted all expenditure data into USD, using the annual average Afghanistan Central Bank official exchange rate for the year 2016 (66.70 Afs per USD). In the multivariate analysis, expenditure variables were converted to their logarithmic form.

For health facility outputs, since the services provided by these health facilities are provided on an ambulatory basis, outpatient visits are selected. Limited inpatient care offered at CHCs was excluded from the analysis. We included the number of visits (by adults and children of all ages) associated with the following conditions in the course of the year: (1) acute respiratory infection, (2) diarrhea, (3) peptic disorder, (4) number of immunizations administered (5) number of antenatal and postnatal visits, and (6) number of all other facility visits. These were the most common visits observed in health facilities for primary care.

These input and output indicators were used to create the health-facilities efficiency scores. The scores range 0–1, where 1 means maximum efficiency. In the multivariate analysis, these scores are the dependent variable. Our explanatory variables are: (1) proportion of facility personnel who are supporting staff (calculated by dividing the number of supporting staff by the number of all facility personnel; (2) proportion of capital costs, from among total annual costs; and (3) province hardship category. The latter is a measure used by the Ministry of Public Health in deciding salary scales and reflects remoteness and the security situation in the province. It consists of four categories. We created three dummy variables, with category one (least hardship category) as the reference category. These variables were selected based on our knowledge of the health system in Afghanistan, which suggested that these factors might influence efficiency, as well as the availability of data.

### Statistical analysis

To start, we conducted a descriptive analysis of all the variables included in the efficiency calculations, as well as the explanatory variables. For continuous variables, means and standard deviations were calculated, while frequencies are shown for categorical variables. We then used ordinary least square regression to assess determinants of variation in efficiency at the health facility level, first by type of health facilities and then by pooling the three types of facilities together. The dependent variables were the facility-specific efficiency scores calculated by the DEA, separated by type of facility and pooled together, for the two analyses respectively. Data were extracted in MS Excel. All statistical analyses were conducted using STATA v.15, except the calculation of the DEA score, which was calculated using R (version 3.5.3).

## Results

### Facility characteristics

In total, 1263 health facilities from 31 provinces were included in the analysis: 272 CHCs, 571 BHCs, and 420 SHCs. Table 1 describes the various variables used to calculate the health-facility efficiency scores, as well as selected explanatory variables. On average, the annual capital expenditure was USD$3908 in CHC, USD$1375 per BHC, and USD$918 per SHC, while the recurrent expenditure was USD$38,601 per CHC, USD$15,680 per BHC and USD$8661 per SHC.

**Table 1:** Description of inputs, outputs and explanatory variables by facility level

Variable	Comprehensive Health Center CHC (n = 272)		Basic Health Center BHC (n = 571)		Sub Health Center SHC (n = 420)	
	Mean Standard Deviation (SD)	Range	Mean Standard Deviation (SD)	Range	Mean Standard Deviation (SD)	Range
Input variables						
Capital cost (USD)	3908 (12,433)	0–133,501	1375 (3011)	0–26,179	918 (1781)	0–15,725
Recurrent cost (USD)	38,601 (25,077)	5855–259,568	15,680 (7679)	231–54,789	8661 (5069)	28–32,521
# Supporting staff	4.57 (1.3)	0–8	1.99 (0.63)	0–14	1 (0.50)	0–10
# Technical staff	10.38 (1.43)	2–18	5.35 (1.02)	1–16	2.47 (0.68)	1–5
Output variables						
# Acute respiratory infection visits	11,590 (5924)	0–39,166	6540 (3586)	653–23,101	3952 (2141)	294–16,426
# Diarrhea visits	42.55 (2417)	0–19,437	2449 (1526)	105–11,500	1521 (908)	9–6452
# Peptic disorder visits	3024 (1579)	0–9933	1705 (985)	101–8478	1123 (632)	69–3962
# Immunization administered	7592 (4132)	580–26,213	4774 (2710)	0–17,778	1053 (1576)	0–10,219
# Antenatal & postnatal visits	6827 (4907)	0–32,286	3155 (2552)	S	1573 (1,265)	0–8072
# Other visits	25,667 (12,898)	400–76,965	13,051 (6864)	622–49,187	7809 (4381)	642–48,044
Explanatory variables						
% Supporting staff	0.3 (0.06)	0–0.47	0.27 (0.06)	0–0.78	0.28 (0.11)	0–0.77
% Capital cost	0.06 (0.13)	0–0.82	0.07 (0.13)	0–0.78	0.09 (0.15)	0–0.85

Values for all input and output variables were most significant for CHCs, followed by BHCs, with SHCs having the smallest input and output values, reflecting the relative sizes of these types of facilities. The means of the explanatory variables were similar across facility types.

### Comparative efficiency separated by type of facilities

**Fig. 1:**
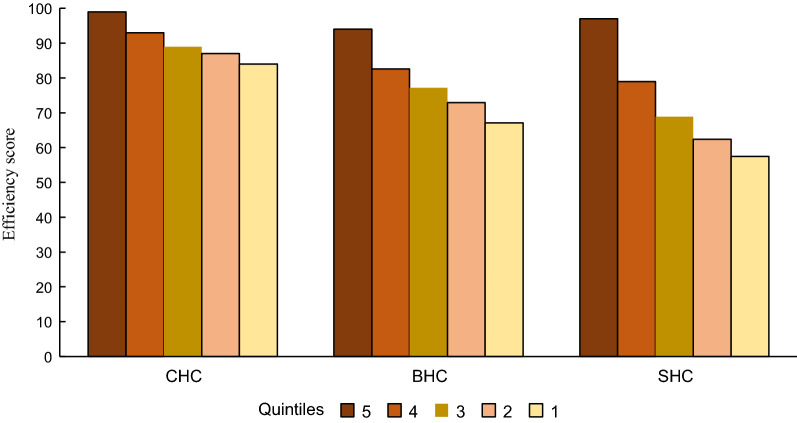
Efficiency scores quintile by health facility level

Figure 1 shows efficiency scores for the three facility types. Since the efficiency score variable is continuous, we created score quintiles, with facilities with the lowest scores in the first quintile, and those with the highest score in the fifth. On average, CHCs, BHCs and SHCs have efficiency scores of 0.90, 0.79, and 0.73 respectively.

In general, CHCs had higher average efficiency scores than BHCs and SHCs. The mean CHC score in the lowest efficiency quintile was fairly high at 0.84, yet there was still room for improvement for facilities in the lowest efficiency quintile up to 0.16 to be fully efficient. In comparison, in BHCs the difference between mean efficiency scores in the highest quintile (0. 94) and the lowest quintile (0.67) was 0.27. For SHCs the difference between highest and lowest quintiles was greatest at 0.40. We can also see that the mean efficiency scores in the highest quintile were fairly similar in all health facilities. The bigger differences were in the bottom two quintiles.

Table 2 shows the ordinary least square regressions of the determinants of efficiency. In BHCs, the explanatory variables were statistically significant, except share of supporting staff. If share of capital cost increases by 1 percentage point, the efficiency would increase by 0.10 percentage points. Additionally, compared to province group 1, groups 2, 3, and 4 had a relatively lower efficiency score, by 4.4, 2.8, and 4.5 percentage points. In SHCs, the share of supporting staff and location were statistically significant. If share of supporting staff increases by 1 percentage point, the efficiency score would be reduced by 0.30 percentage points. Additionally, compared to provinces in group 1, the efficiency in group 2 was significantly lower. The results from the regression model to explain efficiency differences of CHCs. As most CHCs were relatively efficient with an efficiency score between 84 and 99 percent and an average efficiency score of 90 percent. The explanatory variables were not statistically significant, except for the province group 4 and share of supporting staff. A one percentage point increase in the share of supporting staff was associated with 0.18 percentage point’s reduction in efficiency. Additionally, compared to the province group 1, CHCs in province group 4 had a low efficiency by two percent points.

**Table 2:** Regression model of the efficiency at the CHCs, BHCs and SHCs

	CHC		BHC		SHC	
	Coefficient (Standard error)	95% confidence interval	Coefficient (Standard error)	95% confidence interval	Coefficient (Standard error)	95% confidence interval
% Supporting staff	− 0.178*** (0.052)	− 0.28, − 0.076	− 0.067 (0.068)	− 0.201, 0.068	− 0.297*** (0.065)	− 0.426, − 0.169
% Capital cost	0.019 (0.025)	− 0.03, 0.069	0.103*** (0.032)	0.039, 0.165	0.031 (0.047)	− 0.062, 0.124
Hardship province 2	− 0.002 (0.01)	− 0.21, 0.017	− 0.044*** (0.011)	− 0.067, − 0.020	− 0.049* (0.025)	− 0.098, 0.001
Hardship province 3	− 0.008 (0.01)	− 0.027,0.012	− 0.028*** (0.012)	− 0.052, − 0.004	− 0.023 (0.024)	− 0.075 to 0.020
Hardship province 4	− 0.02* (0.011)	− 0.041, 0	− 0.045*** (0.011)	− 0.067, − 0.021	− 0.027 (0.024)	− 0.075,0.020
Constant	0.961*** (0.019)	0.925, 0.998	0.83* (0.019)	0.791,0.868	0.84*** (0.026)	0.788, 0.892

^***^, **, & * denote significance at the *p* < 0.01, *p* < 0.05, & *p* < 0.1 respectively

### Comparative efficiency by pooling all health facilities

When pooling all health facilitates using a single production frontier, we found that the average efficiency score was 0.74 with a standard deviation of 0.25. Out of 1263 health facilities, 102 were on the production frontier, of which 32 were CHCs, 30 BHCs, and 40 SHCs. The efficient health facilities accounted for 8.1% of the total facilities.

**Fig. 2:**
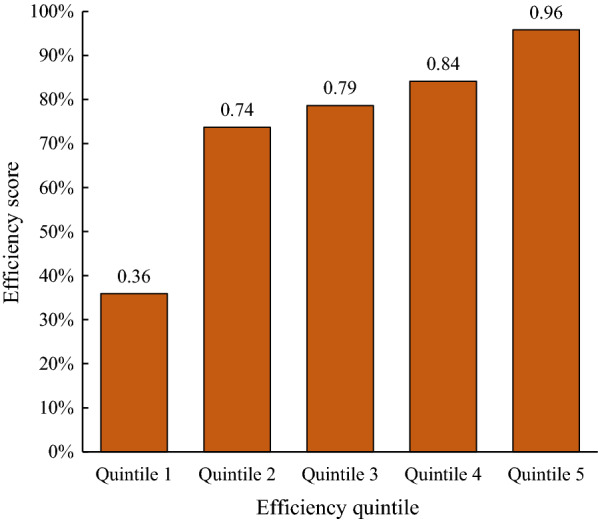
Relative technical efficiency scores from pooled DEA

Figure 2 shows the pooled efficiency scores by quintile. The facilities with the lowest efficiency scores had an average score of 0.36. The average efficiency scores for the remaining quintiles were 0.74, 0.79, 0.84 and 0.96. The average efficiency scores for CHCs, BHCs, and SHCs were 0.81, 0.71 and 0.73, respectively, and the differences were statistically significant (F = 16.12, p < 0.001).

Table 3 provides the results from the regression to assess the determinants of the efficiency scores when pooling all heath facilities. Compared to CHCs, the efficiency score of BHCs and SHCs were lower by 0.11 and 0.07 respectively. Hardship was negatively associated with relative technical efficiency. We also found that higher share of supporting staff was associated with lower relative technical efficiency while higher share of capital costs was associated with higher efficiency. One percentage point increase in supporting staff was associated with a reduction of 0.17 percentage points in efficiency (p < 0.05). One percentage point increase in capital costs was associated with an increase of 0.28 percentage points in efficiency (p < 0.001).

**Table 3:** Determinants of the pooled relative technical efficiency

Pooled efficiency score	Coefficient (Standard error)	T	P > t	95% confidence interval	
BHCs	− 0.108*** (0.018)	− 6.07	0.000	− 0.143	− 0.073
SHCs	− 0.071*** (0.019)	− 3.79	0.000	− 0.108	− 0.034
Hardship province 2	− 0.096*** (0.021)	− 4.58	0.000	− 0.138	− 0.055
Hardship province 3	− 0.141*** (0.021)	− 6.65	0.000	− 0.182	− 0.099
Hardship province 4	− 0.185*** (0.021)	− 8.72	0.000	− 0.226	− 0.143
% supporting staff	− 0.170** (0.086)	− 1.97	0.049	− 0.339	− 0.001
% capita costs	0.357*** (0.051)	7.04	0.000	0.258	0.457
Constant	0.947*** (0.033)	29.1	0.000	0.883	1.011

^***^, **, & * denote significance at the *p* < 0.01, *p* < 0.05, & *p* < 0.1 respectively

## Discussion

This study analyzed the relative technical efficiency of three levels of public primary healthcare facilities in Afghanistan operated by NGOs. We found that the average separate efficiency scores in our study ranged 0.73–0.90 for the different levels. Additionally, different types of health facilities delivered BPHS with varying degrees of efficiency. Providing primary healthcare in CHCs was more efficient than in BHCs and SHCs. Facilities that provide more comprehensive care are more efficient and have limited room for potential efficiency gains. In contrast, lower-level facilities are less efficient and therefore may have more room for improvement inefficiency.

The existence of inefficiency in providing primary health in Afghanistan is consistent with findings from many other countries. Studies conducted in some developing countries report that the majority of primary healthcare facilities are relatively inefficient. For example, a study in Ghana found that 78% of primary health facilities were inefficient with an efficiency score of less than 100%, and about 38% of health facilities had an efficiency score of less than 50% [8]. A study in South Africa determined that 70% of primary healthcare facilities were inefficient with an efficiency score of less than 100% [14]. Our findings show that 60% of BHCs and SHCs have average efficiency scores of 0.70 or less making them relatively inefficient. This suggests that there is room for improve the services delivery if existing resources at health facilities were managed better. In fact, in this study, the difference in efficiency between the lowest and highest efficiency quintile was much broader in facilities that offer fewer services (a range between mean separate efficiency of the highest and lowest quintiles of 0.15 in CHCs, 0.26 in BHCs, and 0.39 in SHCs). The last two offer fewer services than CHCs.

We found a wide range of efficiency scores at the lower-level health facilities (BHCs and SHCs). The relative efficiency score for the lowest quintile of BHCs and SHCs were 0.67 and 0.58 respectively, suggesting that 32.9% and 42.5% of resources could be saved in each facility type while maintaining the same level of care. Our multivariate analysis confirmed these findings. To put this into perspective, in total, USD$7.14 million could be saved had all BHCs and SHCs performed to the level of their most efficient peers. Alternatively, the less efficient health facilities could also increase their outputs with the same level of inputs.

Capital investment in health facilities has been associated with better performance of health facilities in terms of efficiency. This is understandable, especially when the investment is in very needed technologies that will run for many years, once purchased. On the other hand, better-equipped health facilities will serve more consumers due to efficient use of staff time and higher people’s trust in the health facility. However, while supporting staff contribution to the cleanliness and safety of health facilities is essential, their share as a percentage of total health facility expenditure has been associated with lower efficiency. A possible explanation to this finding is lack of involvement of non-technical staff in managing patient flow and patients visits. However, this should be studied further.

A study in Ghana found that health facilities in urban areas were performing relatively more efficiently than rural health facilities [6]. While we did not consider urban vs. rural locations, our findings confirm the importance of geography. Multivariate analysis showed that provinces with higher hardship scores, reflecting among other parameters, remoteness, and more serious security considerations, are working less efficiently. To introduce more precision into estimating health facilities relative technical efficiency scores, more complex calculations might be required, which can be done as expenditure reporting improves and more details become available.

Several limitations of this study should be acknowledged. First, the EMIS collects capital expenditure, but it does not consider depreciation. However, since depreciation is likely to be even among the types of facilities, we do not expect this to affect our findings. Second, the expenditure incurred in the study period (2016 calendar year) may not necessarily have been used to fund service delivery in the same time period, as some health facilities may have procured medicine for 2017 at the end of 2016. However, some facilities may have procured commodities in 2015 that were used in 2016 and were not measured in our data. We expect that these cancel each other, reducing the possibility of bias. Finally, since EMIS data for the health facilities managed by the MoPH was not available, we could not compare their relative technical efficiency within and to the health facilities managed by the NGOs.

## Conclusion

This study shows that higher-level health facilities are relatively more efficient in providing BPHS in Afghanistan and that smaller facilities have more room for improvement. Share of supporting staff and the capital investment in health facilities are associated with the relative technical efficiency of services delivery to the target population. This suggests that there is room to improve the delivery of services if existing resources at health facilities were managed better. A caveat worth noting is that in some cases the purpose of these lower-level facilities is to improve access to the underserved population that would have no access to health services otherwise. Thus, equity can often lead to a trade-off with efficiency goals. While there is always room for efficiency improvement, it is important to consider the broader context that influences the situation and dive deeper into the feasibility of these lower-level facilities to provide more integrated services without compromising their accessibility. Policymakers and NGO program managers can use our findings to examine how to improve relative technical efficiency in using resources in primary health facilities.

## Data Availability

The data is available in the Health Economics and Financing Directorate of the Ministry of Public Health of Afghanistan.
